# Anterior positioning screw in proximal femoral plating restricts posterior tilt of retroverted femoral neck fractures: a retrospective cohort study

**DOI:** 10.1186/s13018-021-02456-9

**Published:** 2021-05-17

**Authors:** Sheng-Bo Nie, Jun-Feng Liu, Jiang-Hua Zhu, Zi-Fei Zhou, Lei Zhang, Long-Po Zheng

**Affiliations:** 1 Department of Orthopedics, Shanghai Tenth People’s Hospital, Tongji University School of Medicine, Shanghai, 200072 China; 2 Department of Orthopedics, Qidong People’s Hospital Affiliated to Nantong University, Qidong, 226200 China; 3 Department of Medical Equipment, Shanghai Tenth People’s Hospital, Shanghai, 200072 China; 4 Shanghai Trauma Emergency Center, Shanghai, 200072 China; 5 Orthopedic Intelligent Minimally Invasive Diagnosis & Treatment Center, Shanghai Tenth People’s Hospital, Tongji University School of Medicine, Shanghai, 200072 China

**Keywords:** Femoral neck fractures, Proximal femoral plating, Posterior tilt, Femoral neck shortening, Positioning screw

## Abstract

**Background:**

Preoperative posterior tilt is a risk factor for fixation failure in femoral neck fractures. This study aimed to evaluate the configuration of anterior positioning screw in proximal femoral plating in the treatment of retroverted femoral neck fractures in terms of resisting posterior tilt.

**Methods:**

We retrospectively analyzed patients with retroverted femoral neck fractures who were fixed by proximal femoral plating from January 2014 to August 2019. All patients were divided into two groups according to screw configuration: anterior long-threaded screw (ALTS, *n* = 36) and normally short-threaded screws (NTS, *n* = 46). Baseline characteristics were reviewed and radiological and clinical outcomes were analyzed. Logistic regression analysis was used to identify risk factors for developing posterior tilt.

**Results:**

Age, gender, Garden classification, posterior comminution, and reduction quality showed no significant difference between the groups. Increased posterior tilt was lower in the ALTS group (3.2°, 2.1–4.3°) than that in the NTS group (5.3°, 4.2–8.3°) (*p* < 0.001), and the percentage of people with > 5° of posterior tilt was also lower in the ALTS group (5, 13.9% vs. 24, 52.2%; *p* < 0.001). Femoral neck shortening (FNS) was lower in the ALTS group (3.1 (2.1–4.7) mm vs. 4.3 (3.1–6.3) mm, *p* = 0.003), though not statistically significant when using 5 mm as the cut-off value. Harris Hip Score in the ALTS group was higher than that in the NTS group (87.0, 84.0–90.0 vs. 82.0, 76.0–84.5; *p* < 0.001). Postoperative complications including delayed union, nonunion, and avascular necrosis were comparable between the groups. Multivariable analysis identified posterior comminution (OR 15.9, 95% CI 3.6–70.3, *p* < 0.001), suboptimal reduction quality (OR 12.0, 95% CI 2.6–56.1, *p* = 0.002), and NTS configuration (reference: ALTS configuration) (OR 21.9, 95% CI 4.1–116.4, *p* < 0.001) as risk factors for developing posterior tilt.

**Conclusions:**

Configuration of anterior positioning screw in proximal femoral plating provides better resistance against posterior tilt in the fixation of retroverted femoral neck fractures. Also, posterior comminution, suboptimal reduction, and NTS configuration (reference: ALTS) are risk factors for developing posterior tilt.

**Trial registration:**

The trial registration number was ChiCTR2000039482.

## Background

The prevalence of femoral neck fractures increases with the aging population and is a serious healthcare problem worldwide due to high morbidity and mortality rates [1]. In internal fixation of femoral neck fractures, fixation failure is not uncommon, and no consensus has been reached regarding the optimal fixation construct [2]. Preoperative posterior tilt (or retroversion) of the femoral head has been recognized as an important risk factor for fixation failure of femoral neck fractures [3–5]. It has been suggested that more retroversion of the femoral head would induce more posterior comminution, which would cause fracture instability [6–8]. If the inclination of posterior tilt is not antagonized, the femoral head would largely develop retroversion after seemingly “good” fixation [8, 9]. Thus, a more specific fixation construct is needed in treating retroverted femoral neck fractures.

Shin et al. [10] reported that posterior fully threaded positioning screw is advantageous for preventing posterior tilt in Garden I and II femoral neck fractures; Zhang et al. [11] reported that inferior two fully threaded compression screws (functions as positioning screws after fixation) can decrease varus deformity and fixation failure in vertical femoral neck fractures. However, to the best of our knowledge, no studies have been reported focusing on hybrid screw-plating construct for the treatment of retroverted femoral neck fractures, and whether using an anterior positioning screw in a plating construct can resist posterior tilt remains unknown.

In this study, we retrospectively analyzed a novel configuration of anterior long-threaded positioning screw in proximal femoral plating for the fixation of retroverted femoral neck fractures. Specifically, we mainly evaluated its effects in resisting posterior tilt and femoral neck shortening. Additionally, risk factors for developing posterior tilt of femoral head were also identified using multivariable analysis.

## Methods

### Study design and patient selection

This study was conducted in accordance with the Declaration of Helsinki. The requirement for informed consent from the patients (just for this observational study) was waived due to the retrospective nature of this study, and the current study was approved by the ethics committee of our hospital. The inclusion criteria were as follows: (1) femoral neck fractures with preoperative posterior tilt, (2) closed reduction and proximal femoral plate fixation (anterior long-threaded cannulated screw (ALTS) configuration or normally short-threaded cannulated screws (NTS) configuration), and (3) a minimum follow-up of 12 months. The exclusion criteria were more than 10° of remaining posterior tilt after reduction, pathological fractures, malignancies, hip arthritis, or loss to follow-up. Finally, a total of 82 patients were included in this study (ALTS, *n* = 36; NTS, *n* = 46). The medical records were reviewed and baseline characteristics were compared between the two groups.

### Fracture management

In the ALTS group, the patient was placed in a supine position under general anesthesia. Fracture reduction was performed by internal rotation of the affected lower extremity and manual pressure on the anterior part of femoral neck [12]. A K-wire that was inserted to the femoral head was used to assist reduction. After satisfactory reduction was achieved, the plate was placed and three inverted guide pins were inserted through the plate holes in the configuration of inferior-center (along the medial cortex of femoral neck), superior-anterior, and middle-posterior [12]. Then, a short-threaded screw was inserted along the inferior-center guide pin, followed by insertion of a long-threaded positioning screw along the superior-anterior guide pin. Then, another short-threaded screw was inserted posteriorly along the corresponding guide pin and rational compression was imposed to achieve instant bone contact at the fracture site. The plate was finally fixed to the femoral shaft with a distal screw. In the NTS group, the operations were all the same except that a short-threaded compression screw was used superior-anteriorly. Non weight-bearing exercise started instantly after surgery. Partial weight-bearing with walker assistance was allowed when radiological evidence of fracture healing is confirmed (at least 1 month after discharge). Full weight-bearing is allowed when fracture union is confirmed on the follow-up X-rays (three months or later).

### Clinical and radiological outcome measurement

**Fig. 1:**
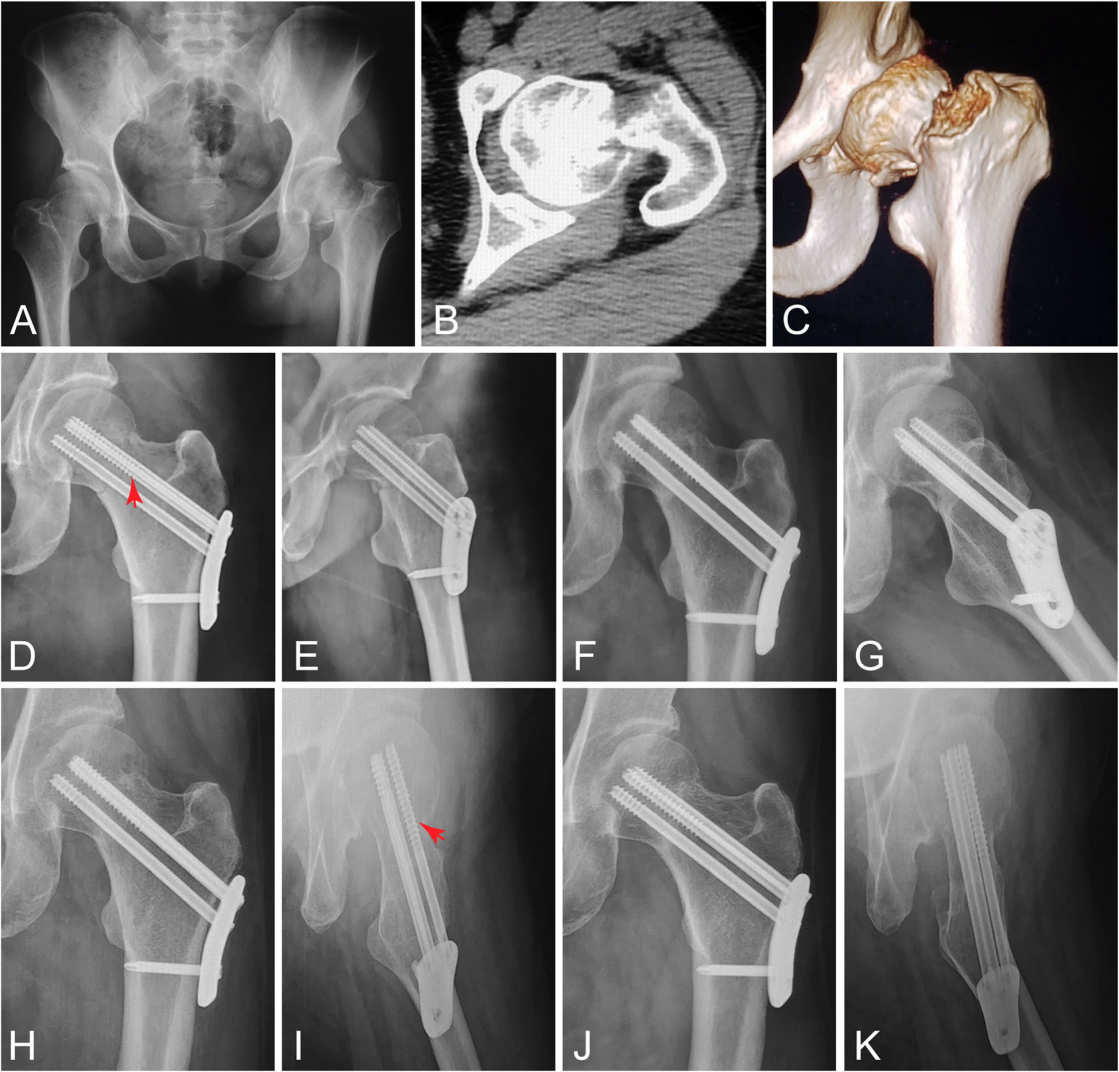
Measurement of CCD angle (**a**), amount of femoral neck shortening (**b**), and posterior tilt angle (**c**) in femoral neck fractures. CCD, caput-collum-diaphysis

Medical records and radiographs were reviewed for the following data: age, gender, Garden type, posterior comminution, and reduction quality. Smooth contact of the medial cortex was defined as excellent reduction, translation of less than one cortex was defined as good, while more than one cortex thickness of residual translation was regarded as moderate/poor. The caput-collum-diaphysis (CCD) angle was measured as described by Park et al. [12]. Briefly, a line from the center of the femoral head to the center of the femoral neck constitutes the femoral neck axis. The angle between the femoral neck axis and the bisecting line of the femoral shaft constitutes the CCD angle (Fig. 1). The amount of femoral neck shortening (FNS) was measured on AP radiographs as described by Zlowodzki et al. [13]. In short, the contour of the unaffected side was overlapped on the contour of the injured side. Changes in the *x*-axis (termed *x*) and *y*-axis (termed *y*) were measured. The amount of FNS at the angle of the femoral neck (termed *z*) was calculated using *θ* as the corresponding CCD angle as follows: *z* = *y*·sin(*θ*) + *x*·cos(*θ*), as was described by Weil et al. [14]. Decreased CCD angle was calculated by comparing CCD angle measured at 12 months postoperatively with that of the unaffected side measured before surgery. Posterior tilt was measured using the method as described in the literature [6]. Nonunion was defined as a clear fracture line on the X-ray at follow-up of 6 months [15]. Harris Hip Score (HHS) was assessed at follow-up of 12 months [16].

### Statistical analysis

For continuous data, normality was tested using the Kolmogorov-Smirnov test. Normally distributed continuous data were presented as mean ± standard deviation (SD), while median and interquartile range were presented for non-normally distributed continuous data. Categorical variables were presented as frequencies or percentages. Independent sample *t* test was used to compare normally distributed continuous data; otherwise, non-parametric test was conducted. Chi-square test was performed for categorical variables. Statistical significance was defined as *p* < 0.05. Univariate logistic regression was performed on each selected variable to determine differences between the two groups. Odds ratios were obtained with 95% confidence intervals. Variables with *p* ≤ 0.05 were incorporated into the subsequent multivariate analysis. Statistical analyses were performed using IBM SPSS (version 22.0) and GraphPad Prism (version 8.0).

## Results

The mean follow-up time was 14.0 (12.0–16.0) months for the ALTS group and 15.5 (12.8–16.0) months for the NTS group (*p* = 0.49). The two groups were comparable regarding age, gender, Garden type, posterior comminution, and reduction quality (*p* > 0.05, Table 1).

**Table 1:** Baseline characteristics and comparative analysis between the two groups

Variables	ALTS (*n* = 36)	NTS (*n* = 46)	*p* value
Age (years)	56.3 ± 9.4	54.6 ± 7.6	0.37
Gender (male/female)	15/21	23/23	0.45
Garden type			0.33
I/II	13	12	
III/IV	23	34	
Posterior comminution	13	18	0.78
Reduction quality			0.95
Excellent/good	24	31	
Moderate/poor	12	15	
Increased posterior tilt (°)	3.2 (2.1–4.3)	5.3 (4.2–8.3)	< 0.001*
Increased posterior tilt (> 5°)	5 (13.9%)	24 (52.2%)	< 0.001*
Femoral neck shortening (mm)	3.1 (2.1–4.7)	4.3 (3.1–6.3)	0.003*
Femoral neck shortening (> 5 mm)	8	18	0.10
Decreased CCD angle (°)	3.4 (1.9–4.3)	3.5 (2.4–5.4)	0.30
Harris Hip Score	87.0 (84.0–90.0)	82.0 (76.0–84.5)	< 0.001*
Delayed union	6	7	0.86
Nonunion	2	3	0.86
Avascular necrosis	4	6	0.79
THA	5	7	0.87

*ALTS* anterior long-threaded cannulated screw, *NTS* normally short-threaded cannulated screws, *THA* total hip arthroplasty

*Statistically significant

### Radiological and clinical outcomes

**Fig. 2:**
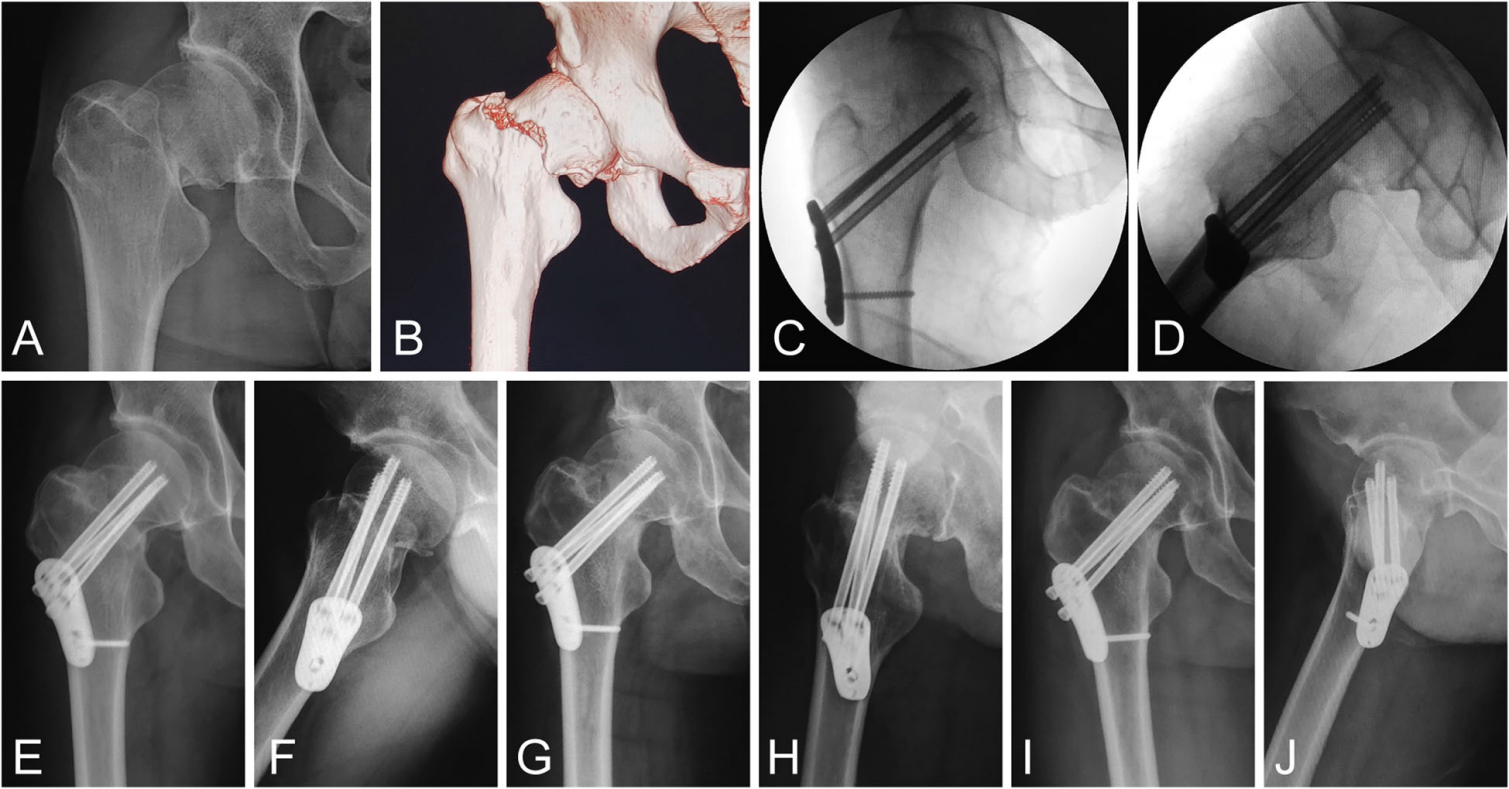
Proximal femoral plating of a 41-year-old female with retroverted femoral neck fracture (Garden type III). **a** Preoperative X-ray. **b**, **c** Preoperative CT scans and three-dimensional reconstruction. Postoperative radiographs at 1 day (**d**, **e**) (arrowhead: anterior long-threaded screw), 4 months (**f**, **g**), 6 months (**h**, **i**) (arrowhead: anterior long-threaded screw), and 12 months (**j**, **k**) (posterior tilt: − 3.3°)

Figure 2 shows a representative case of retroverted femoral neck fracture fixed by proximal femoral plating using ALTS configuration. A long-threaded cannulated screw was inserted anteriorly (Fig. 2d). This patient achieved uneventful bone union six months postoperatively (Fig. 2h, i). Specifically, at follow-up of 12 months, barely any increased posterior tilt (− 3.3°), decreased CCD angle (3.8°), or FNS (2.6 mm) was observed. The HHS at final follow-up was excellent.

**Fig. 3:**
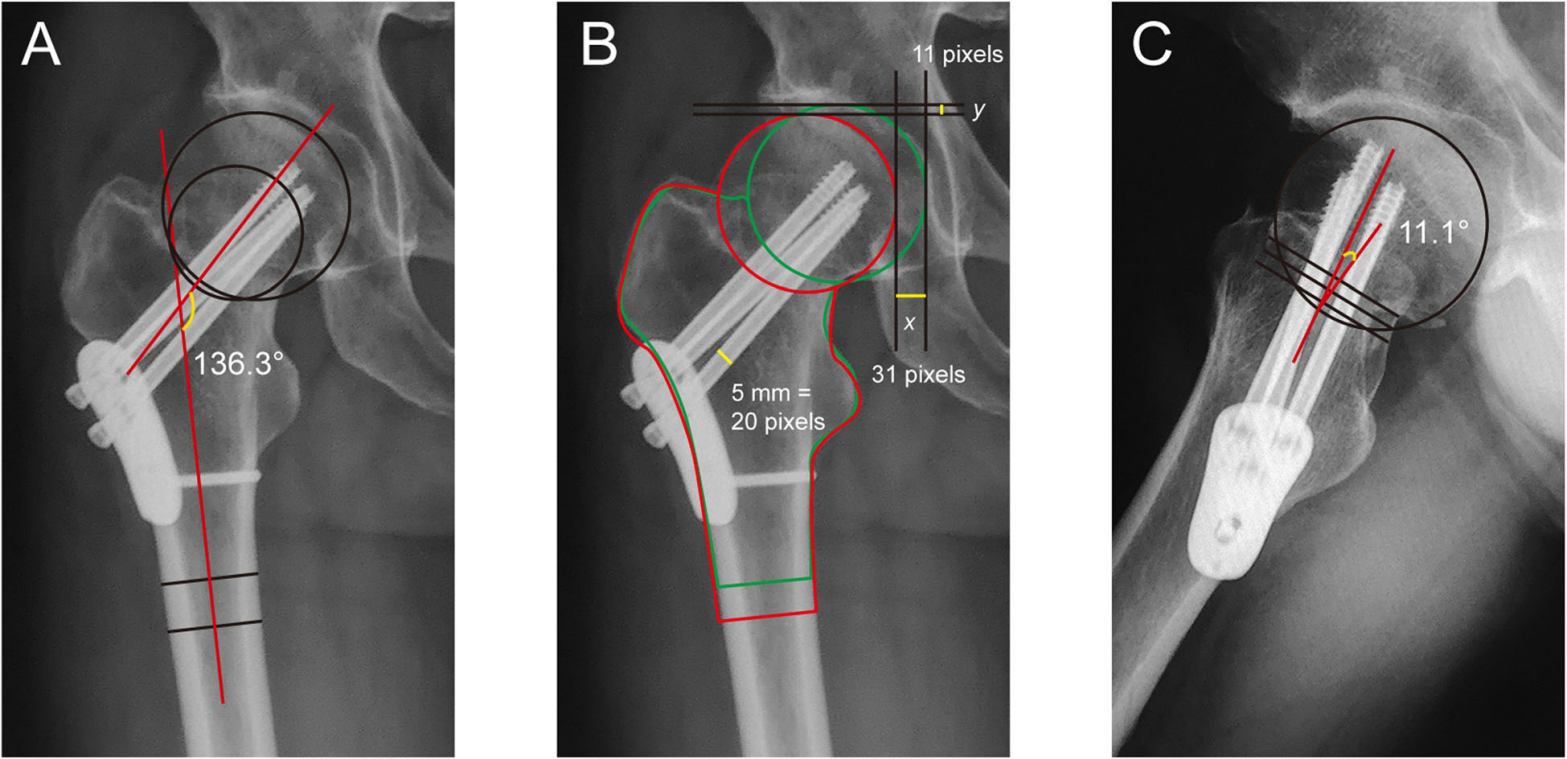
Proximal femoral plating of a 53-year-old male with retroverted femoral neck fracture (Garden type III). **a** Preoperative X-ray. **b** Three-dimensional reconstruction of CT scans. **c**, **d** Intraoperative anteroposterior and lateral radiographs of definitive fixation (posterior tilt: 4.7°). Postoperative radiographs at 1 month (posterior tilt: 11.1°) (**e**, **f**), 6 months (**g**, **h**), and 12 months (**i**, **j**)

Another representative case of retroverted femoral neck fracture treated with proximal femoral plating using NTS configuration is shown in Fig. 3. This patient achieved good reduction, while obvious posterior tilt occurred at follow-up of one month (Fig. 3e, f), and finally, this patient developed malunion, with increased posterior tilt being 6.4°, FNS being 7.2 mm, but with no significant varus deformity (decreased CCD angle: − 5.8°). The HHS was 76 and this patient received no further treatment due to endurable function.

**Fig. 4:**
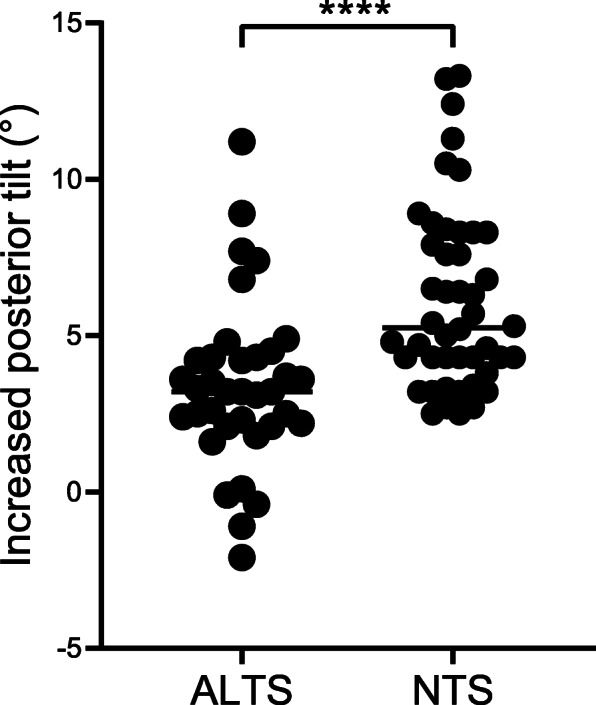
Comparative analysis of increased posterior tilt between the two groups. **** *p* < 0.0001. ALTS, anterior long-threaded cannulated screw; NTS, normally short-threaded cannulated screws

In total, increased posterior tilt was significantly lower in the ALTS group (3.2°, 2.1–4.3°) than that in the NTS group (5.3°, 4.2–8.3°) (*p* < 0.001, Table 1, Fig. 4), and the percentage of people with > 5° of posterior tilt was also significantly lower in the ALTS group than that in the NTS group (5, 13.9% vs. 24, 52.2%; *p* < 0.001). FNS was significantly lower in the ALTS group than that in the NTS group (3.1, 2.1 – 4.7 mm vs. 4.3, 3.1–6.3 mm; *p* = 0.003), though when using 5 mm as the cut-off value [12], the difference showed no statistical significance (*p* = 0.10). Harris Hip Score in the ALTS group was higher than that in the NTS group (87.0, 84.0–90.0 vs. 82.0, 76.0–84.5; *p* < 0.001). Amount of decreased CCD angle, rate of delayed union, nonunion, avascular necrosis, and number of patients who converted to total hip arthroplasty (THA) all showed no significant difference (*p* > 0.05).

### Bivariate analysis of potential risk factors for developing posterior tilt (> 5°)

To explore the potential risk factors for developing posterior tilt after surgery in retroverted femoral neck fractures, we reclassified all patients according to angle of increased posterior tilt with the cut-off value being 5° (Table 2). Age, gender, Garden type, posterior comminution, fixation type, and reduction quality were compared. As a result, posterior comminution, NTS configuration (reference: ALTS configuration), and reduction quality showed a significant difference between the two groups (*p* < 0.05), which could be regarded as potential risk factors for developing posterior tilt.

**Table 2:** Bivariate analysis of factors for developing posterior tilt (> 5°)

Variables	Increased posterior tilt (≤ 5°) (*n* = 53)	Increased posterior tilt (> 5°) (*n* = 29)	*p* value
Age (years)	55.3 ± 8.2	55.5 ± 8.9	0.94
Female gender	28	16	0.84
Garden type III/IV	35	22	0.36
Posterior comminution	11	20	< 0.001*
NTS configuration	22	24	< 0.001*
Reduction quality (moderate/poor)	10	17	< 0.001*

*Statistically significant

### Univariable and multivariable analysis of risk factors for developing posterior tilt (> 5°)

To further identify risk factors for developing posterior tilt, we established a logistic regression model containing posterior comminution, reduction quality, and NTS configuration (reference: ALTS) (Table 3). Univariable analysis revealed that posterior comminution, reduction quality, and NTS configuration were statistically significant. Subsequent multivariable analysis identified posterior comminution (OR 15.9, 95% CI 3.6–70.3, *p* < 0.001), reduction quality (moderate/poor) (OR 12.0, 95% CI 2.6–56.1, *p* = 0.002), and NTS configuration (reference: ALTS) (OR 21.9, 95% CI 4.1–116.4, *p* < 0.001) were risk factors for developing posterior tilt.

**Table 3:** Univariable and multivariable logistic regression analysis of risk factors for developing posterior tilt (> 5°)

Variables	Univariable analysis		Multivariable analysis	
	OR (95% CI)	*p* value	OR (95% CI)	*p* value
Posterior comminution	8.5 (3.0–23.8)	< 0.001*	15.9 (3.6–70.3)	< 0.001*
Reduction quality (Moderate/poor)	6.1 (2.2–16.7)	< 0.001*	12.0 (2.6–56.1)	0.002*
NTS configuration	6.8 (2.2–20.5)	0.001*	21.9 (4.1–116.4)	< 0.001*

*OR* odds ratio, *CI* confidence interval

*Statistically significant

## Discussion

Posterior tilt/posterior neck collapse is frequently encountered after fixation of femoral neck fractures and is associated with further neck shortening and nonunion [17, 18]. Additionally, a preoperative posterior tilt of more than 20° was reported to be a significant predictor for reoperation [6]. It is likely that preoperative posterior tilt of femoral head would largely damage the bony mechanical transduction of the posterior cortex. Additionally, the inclination of femoral head retroversion is less likely to be counteracted with the use of normal fixation construct (parallel partially threaded screws), especially in the presence of posterior comminution [19, 20]. Thus, a more specialized fixation construct is needed to resist posterior tilt in retroverted femoral neck fractures.

**Fig. 5:**
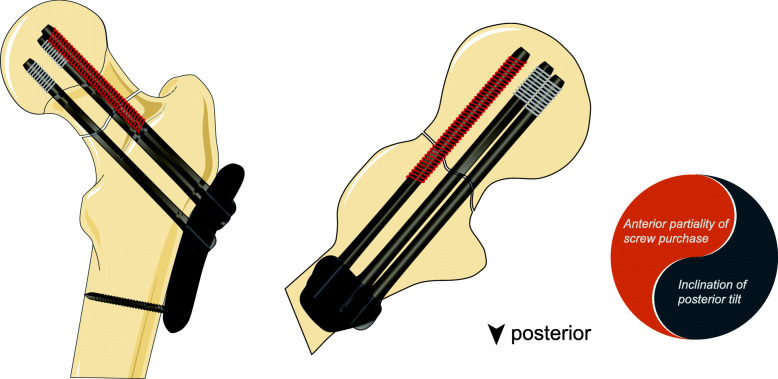
Graphical illustration of proximal femoral plating with an anterior long-threaded cannulated screw in the fixation of retroverted femoral neck fractures. By employing a screw with longer thread in the anterior part of femoral head, which provide greater holding force to avoid malposition, inclination of posterior tilt of the femoral head could be better counteracted

Shin et al. [10] recently reported that in the classic configuration of parallel cannulated screws, replacing a partially threaded cannulated screw with a posterior fully threaded positioning screw can prevent femoral neck shortening and posterior tilt in Garden I and II femoral neck fractures. This hybrid construct was regarded to be more length- and angle-stable, as was demonstrated in a previously biomechanical study [21]. Similarly, in our study, the anterior partially threaded compression screw was replaced with a long-threaded positioning screw (Fig. 5), which would also provide length- and angle-stability to prevent posterior tilt. Just as was shown in our results, lower increased posterior tilt and smaller amount of FNS were observed in the ALTS group than that in the NTS group (Table 1). In addition, lateral plating offers better integral property by combining the three screws into a whole one construct. What’s more, the screw purchase of the anterior long-threaded screw was larger than that of the other two screws, which was helpful to counteract the tendency of posterior tilt of femoral head.

There might be concerns of inducing nonunion by replacing compression screws with non-sliding positioning screws. However, a study compared short- and long-threaded cancellous screws in the fixation of femoral neck fractures in a randomized trial of 432 patients, and no difference was found regarding fracture healing complications [22]. Thus, we speculate that the absolute length of screw thread may not be determinant in femoral neck fracture fixation. Other factors, including fracture geometry (which largely determines the tendency of fracture displacement), patient characteristics, and reduction quality, may be more important. In this perspective, the strength of our study is that we focused on femoral neck fractures that presented with preoperative posterior tilt (in consideration of homogeneity of this study). In these patients, femoral head retroversion would largely occur postoperatively due to damaged posterior cortical transduction. In this situation, anterior partiality of screw purchase in the ALTS configuration was demonstrated to significantly resist posterior tilt of femoral head. Consequently, we hold the view that in the fixation of femoral neck fractures, implant construct with biomechanical partiality that specifically counteracts the inclination of fracture displacement is advantageous, which could help to create a balanced mechanical environment for fracture healing (Fig. 5).

Chiang et al. [23] recently reported that three fully threaded headless compression screws, which were normally regarded as a non-sliding length-stable construct, failed to prevent FNS and varus collapse in Garden I and II femoral neck fractures compared with partially threaded screws. We think that although the patient population were all non-displaced fractures, the tendency of fracture displacement (in three-dimensional orientation) may not be the same due to potential heterogeneity regarding fracture geometry, bone quality, etc. For this reason, the universally used three fully threaded screws may not be effective in all patients. In comparison, the ALTS construct in our study with anterior partiality of screw purchase was used to treat retroverted femoral neck fractures (which was more targeted and specific), and yielded favorable results in terms of decreasing posterior tilt of the femoral head. However, precise prediction and evaluation of three-dimensional stability of femoral neck fractures (or inclination of displacement) is still a difficult task, which deserves further study in the future.

FNS was also frequently encountered after fixation of femoral neck fractures. Of note, FNS was reported to be related with length discrepancy of the lower extremity, decreased abductor length, femoral head collapse, hip impingement, and inferior hip function [24, 25]. Worse still, femoral neck compaction after surgery has been reported as an important risk factor for avascular necrosis [25]. In our study, the ALTS configuration showed a statistically significant difference in decreasing the amount of FNS in the fixation of retroverted femoral neck fractures. One reason may be that by creating a more balanced biomechanical environment, further collapse of the femoral neck would be largely hampered. Unfortunately, when using 5 mm as the cut-off value, the difference was not significant, which implies limited clinical significance. Small sample size and specific study design (anteriorly-partialized screw purchase to resist posterior tilt) may be two explanations for this. As to decreased CCD angle between the two groups, no significant difference was found in our results, which implies that the ALTS configuration has minimal effect in terms of resisting varus deformity of the femoral head. A more specific construct is needed to achieve this purpose. Regarding the difference of HHS between the two groups, given that the minimal clinically important difference was estimated to be around eight points in young population [26], the mean HHS difference of five points at follow-up of 12 months may not be clinically significant. This may be due to the retrospective nature of this study and the small sample size. However, given that posterior tilt is recognized as an important risk factor for fixation failure [9], and the risk of increased posterior tilt > 5° was significantly lower in the ALTS group than in the NTS group, we think this finding would be clinically significant.

Regarding the risk factors for developing posterior tilt of femoral head, we identified posterior comminution as a risk factor, which implies that posterior bony transduction is of vital importance to achieve fracture stability; NTS configuration (in reference to ALTS configuration) was also identified as a risk factor, which proved the superiority of the ALTS configuration in resisting femoral head retroversion; also, inferior reduction quality was identified as a risk factor, which was in line with the literature [27]. Altogether, anterior partiality of screw purchase that specifically counteracts the inclination of retroversion and good reduction quality was important for achieving favorable outcomes.

Several limitations existed in this study. First, this retrospective study may contain potential selection bias. Second, the sample size in each group is relatively small. Third, a minimum follow-up of 12 months is relatively short, and long-term complications remain to be evaluated.

## Conclusions

In retroverted femoral neck fractures, the anterior positioning screw in proximal femoral plating provides better resistance against femoral head retroversion. In addition, posterior comminution, suboptimal reduction, and NTS configuration (reference: ALTS) are risk factors for developing posterior tilt.

## Data Availability

The datasets used and/or analyzed during the current study are available from the corresponding author on reasonable request.

## Abbreviations

ALTS: Anterior long-threaded cannulated screw
CCD: Caput-collum-diaphysis
CI: Confidence interval
FNS: Femoral neck shortening
HHS: Harris Hip Score
NTS: Normally short-threaded cannulated screws
OR: Odds ratio
SD: Standard deviation
THA: Total hip arthroplasty.
